# Cremation

**Published:** 1874-05

**Authors:** 


### Editorial.

ALTHOUGH the subject of *Cremation*, which is engaging much attention just now, not only from the medical and scientific journals of the day, but also from the more ephemeral issues of the newspaper press, does not very nearly concern the majority of our readers, still the subject is one which involves certain scientific principles and sanitary bearings which must be weighed and determined by the profession to which we belong.

The proposition to reduce the organic materials of the lifeless human body back to their more elemental gaseous forms of carbonic acid, water and ammonia by means of fire, and to garner up and preserve in a memorial way the more indestructible earthy and phosphatic ashes of the dead, is by no means new. "This custom is well known to have prevailed among most of the Eastern nations, and continued with their descendants after they had peopled the different parts of Europe. Hence we find it prevailing in Greece, Italy, Gaul, Brittain, Germany, Sweden, Norway, and Denmark, until the introduction of christianity abolished it." The funeral pyre still exists amongst some of the oriental tribes of our own day.

With the progressive increase of population upon the globe, with the rapid march of science, of art, and of mechanical invention, and the aggregation of continually augmenting masses of human mortality upon restricted areas of earth's surface, whereby the living and the dead are made to dwell together in close proximity generation after generation, certain inconveniences are being felt to result in densely populated sections which call for remedy.

The method of sepulture almost universally observed by the inhabitants of christian nations involves the gradual decomposition of the organized structures of the human body and re-arrangement of chemical elements into new compounds which, though fitted in the order of nature's laws, for the sustentation and thrift of vegetable life and growth, are found to be pernicious and destructive in their effects upon the living animal organism of the species from which they have been derived. These hurtful products of retrograde metamorphosis may go off into the atmosphere and be inhaled into the lungs, and more or less absorbed into the vital blood, or passing by absorption into the surrounding earth become dissolved in the waters which permeate beneath the surface everywhere, and aggregating find issue in wells, springs and larger streams from which supplies are daily drawn for the sustentation of the populace, and thereby literally putting death in the pot from which is to be drawn the materials for the development and continuance of life.

This subject, little as it may individually concern us and ours, is nevertheless becoming one of the most *vital* character to the inhabitants of certain densely populated cities and countries. Whilst the ancient methods of embalming the dead with spices and balsamic resins would render the process of inevitable decay so slow and gradual as to wholly prevent any serious impregnation of the surrounding atmosphere with noxious gasses, and the modern and elegant air-tight and hermetically sealed casket would effectually stay the progress of decomposition, and atmospheric contamination, until accident, or some convulsion of nature, should crack the iron shell and enable grim death to claim at last the complete fruits of his victory over frail mortality; still these human devices to stay for a season the execution of the divine fiat "dust thou art and unto dust thou shalt return," are but for the few, and the millions of the great "unwashed" must ever remain, under our present system, food for the worms. In this aspect of the case it has been proposed to bring to our aid the resources of modern chemical and pyrotechnic art to hastily unlock and re-arrange the links of chemical affinity so as to give to the hungry world of vegetation its coveted pabulum, and to the heart-stricken mourners their little urn of treasur-able human ashes.

From a scientific stand-point, it has been urged that it is not only a duty to guard the living against the slow but sure poisoning by juxtiposition with the mouldering dead, but likewise a duty to make economic use of the fertilizing resultants of the cremation process for horticultural and agricultural ends. From a religious stand-point it might be questioned whether it be consistent to longer lift our puny arms in rebellion against the divine purpose in our mortality and dissolution, or to essay by our art to interrupt, though it be for a brief season, the revolution of the great chemical cycle which is continually feeding to the vegetable creation the effete elements of the animal, that the former may, in its turn, furnish food for the latter. And, moreover, from an æsthetic stand-point, might it not be urged that the contemplation of our loved and lost transformed into the blushing rose or the pale delicate spathe of the modest lilly, or even into the gnarled oak whose staunch ribs bear upon the great deep a nation's navy, or indeed into the great staff which supports the life of the race, is infinitely more elevating and satisfying than the mental picture of an ever moving mass of countless entymological hyænas preying upon what little remains to remind us for but a fleeting day of our cherished loved ones.

Pretermitting any expression of individual preference in the matter of sepulture, we close the hasty contemplation of this fruitful subject with the remark, "*chacun a son gout.*"

